# Rethinking Integration of Environmental and Behavioral Stressors; Back to Energy Homeostasis and Function

**DOI:** 10.1093/function/zqab074

**Published:** 2022-01-08

**Authors:** Patricia E Molina

**Affiliations:** Department of Physiology, Louisiana State University Health Sciences Center, New Orleans, LA 70112, USA

At this crucial point, as we emerge from the COVID-19 pandemic and transition to an endemic state, it is imperative that we refocus our biomedical research priorities. The pandemic was devastating in terms of human life and economic health, and brought about significant collateral damage that now poses cumulative health burden on society. Among the existing knowledge underscored and emphasized is the importance of environmental interactions with host factors in determining risk for enhanced morbidity and mortality, in this case from an infectious challenge. The recognition of the dynamic viral traits and the differential susceptibility and unpredictable response to SARS-CoV-2 infection beckons us to reconsider how our science must continuously adapt to consider the multiplicity of factors impacting Function. It also is a moment to reflect on how diet and substance use are impacted by social isolation, stress, and anxiety, and how the consequent psychosocial burden can in turn impact disease pathogenesis.

**Figure 1.**
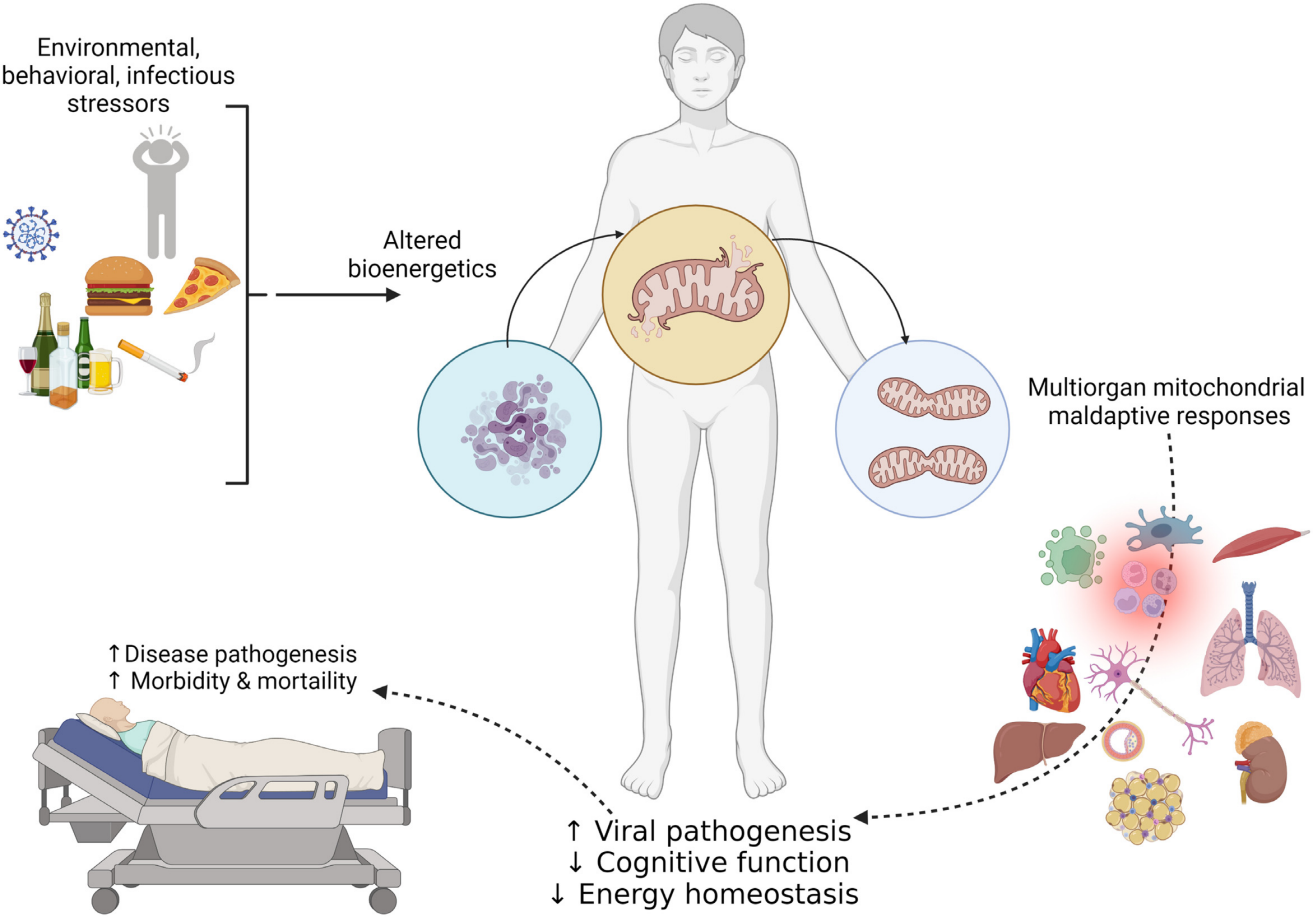
Environmental, behavioral, and infectious stressors challenge the cellular mechanisms involved in maintaining energy homeostasis. The increased energy demands and shifts in metabolic pathways exerts an increased demand on enzymatic and mitochondrial components, increasing the frequency of maladaptive responses in multiple organ systems. The impaired bioenergetics leads to increased viral pathogenesis, and associated comorbidities including decline of cognitive function and whole-body energy homeostasis. Together, these added stressors increase disease pathogenesis, morbidity, and mortality from infectious challenges. Created with Biorender.

From the onset, underlying comorbidities were identified to be predisposing factors for symptomatic and severe SARS-CoV-2 infection .^1^ This is not surprising, as noncommunicable diseases, including heart disease, stroke, cancer, diabetes, and chronic lung disease, are collectively responsible for almost 70% of worldwide mortality.^2^ The primary drivers for these conditions are tobacco use, physical inactivity, unhealthy alcohol use, and low dietary quality (Figure 1). The frequency of anxiety and depression experienced among U.S. adults increased significantly during the pandemic,^3^ and this was frequently associated with unhealthy alcohol use compounding the overall burden on health.^4^

Unhealthy alcohol use is the leading cause of preventable death in the United States, and more than 50% of alcohol-attributable deaths and years of potential life lost are due to chronic comorbidities.^5^ Alcohol affects multiple organ systems directly, and indirectly.^6^ Increasingly, alcohol's impact on energy homeostasis ranging from cellular energetics to glucose homeostasis is being recognized.^7^ Our group's current view is that alcohol-induced alterations in bioenergetics may be a linchpin underlying pathophysiological responses to increased energy demands imposed by environmental stressors. This can be illustrated by alcohol-induced alterations in immune cell energetics and the resulting alterations in host response to infection, leading to decreased capacity for bacterial and viral clearance and dysregulated production of cytokines and chemokines that aid in the resolution of an infectious challenge.^8^ In addition, alcohol can directly or indirectly prime immune cell metabolism dysregulating or preventing the homeostatic shifts in energy substrate preference needed to support proper cellular responses to sterile or non-sterile cellular challenges.

At the core of maintaining energy homeostatic flexibility is the cellular enzymatic and mitochondrial structural and functional integrity. Maintenance of mitochondrial homeostasis involves transcriptional regulation of mitochondrial biogenesis through the action of the PGC-1 family of co-activators that respond to changes in nutrient status and environmental signals.^9^ In immune cells, a dynamically regulated shift between glycolysis and oxidative phosphorylation driven by chemotactic or pathogenic stimuli specific to the underlying challenge or stress, is critical to sustaining the increased metabolic demands associated with phagocytosis, cytokine production, chemotaxis, and cell proliferation. Interestingly, obesity—a condition that epitomizes energy homeostasis dysregulation—is among the most prevalent risk factors for morbidity and mortality from SARS-CoV-2 infection, an association that warrants further understanding of predisposing cellular defects. Though recognized for decades as the cellular organelle serving as the powerhouse, renewed interest in mitochondrial homeostasis has been elicited by recognition that defective mitochondrial structure and function are associated with malignancies, metabolic instability, and neurocognitive and neurodegenerative diseases.^10^ Improved genetics, imaging, and big data approaches promise a renewal of research efforts to elucidate the cellular adaptive responses as they pertain to shifting energetic demands triggered by cellular challenges and identification of a causal link between cellular energy dyshomeostasis and abnormal cellular responses, including those to invading pathogens.

Future research should be influenced by our recognition of the intersection of environmental factors with physiological function, and how the health consequences of the pandemic unraveled previously unsuspected relationships and interactions. This is particularly so for diet, alcohol, and social stress.
